# Regional differences of the sclera in the ocular hypertensive rat model induced by circumlimbal suture

**DOI:** 10.1186/s40662-022-00319-w

**Published:** 2023-01-04

**Authors:** Mingfang Xia, Endong Zhang, Fei Yao, Zhaohua Xia, Mingmin Zhou, Xufang Ran, Xiaobo Xia

**Affiliations:** 1 Eye Center of Xiangya Hospital, Central South University, Changsha, 410008 Hunan China; 2 Hunan Key Laboratory of Ophthalmology, Changsha, 410008 Hunan China; 3 National Clinical Research Center for Geriatric Disorders, Xiangya Hospital, Central South University, Changsha, 410008 Hunan China; 4 Department of Ophthalmology, People’s Hospital of Ningxia Hui Autonomous Region, Yinchuan, 750004 Ningxia China

**Keywords:** Sclera, Axial length, Glaucoma, Retinal ganglion cells, Animal models

## Abstract

**Purpose:**

To describe the regional differences of the sclera in ocular hypertension (OHT) models with the inappropriate extension of the ocular axis.

**Methods:**

To discover the regional differences of the sclera at the early stage, OHT models were established using circumlimbal suture (CS) or sclerosant injection (SI). Axial length (AL) was measured by ultrasound and magnetic resonance imaging. The glaucoma-associated distinction was determined by intraocular pressure (IOP) and retrograde tracing of retinal ganglion cells (RGCs). The central thickness of the ganglion cell complex (GCC) was measured by optical coherence tomography. RGCs and collagen fibrils were detected using a transmission electron microscope, furthermore, anti-alpha smooth muscle actin (αSMA) was determined in the early stage after the operation.

**Results:**

Compared with the control group, the eyes in OHT models showed an increased IOP (*P* < 0.001 in the CS group, *P* = 0.001 in the SI group), growing AL (*P* = 0.026 in the CS group, *P* = 0.043 in the SI group), reduction of central RGCs (*P* < 0.001 in the CS group, *P* = 0.017 in the SI group), thinning central GCC (*P* < 0.001 in the CS group), and a distinctive expression of αSMA in the central sclera in the early 4-week stage after the operation (*P* = 0.002 in the CS group). Compared with the SI group, the eye in the CS group showed a significantly increased AL (7.1 ± 0.4 mm, *P* = 0.031), reduction of central RGCs (2121.1 ± 87.2 cells/mm^2^, *P* = 0.001), thinning central GCC (71.4 ± 0.8 pixels, *P* = 0.015), and a distinctive expression of αSMA (*P* = 0.005). Additionally, ultrastructural changes in RGCs, scleral collagen fibers, and collagen crimp were observed in the different regions. Increased collagen volume fraction in the posterior segment of the eyeball wall (30.2 ± 3.1%, *P* = 0.022) was observed by MASSON staining in the CS group.

**Conclusion:**

Regional differences of the sclera in the ocular hypertensive rat model induced by CS may provide a reference for further treatment of scleral-related eye disorders.

**Supplementary Information:**

The online version contains supplementary material available at 10.1186/s40662-022-00319-w.

## Background

The sclera, one of the main components of the eyeball wall, is important to vision, especially in myopia and glaucoma [1]. Clinical evidence has shown that the loss of the visual field is more significant in patients with primary open-angle glaucoma (POAG) with elongated axial length (AL) [2–4]. Tun et al. reported that the anterior surface of the peripapillary sclera (PPS) of the elderly adult population is a v-shaped configuration with a deep cup, however, the retina was not measured [5]. The change in shape with age may have an impact on the biomechanical environment of the optic nerve head (ONH). The current understanding of the characteristics and mechanisms of postnatal AL growth and development of myopia or glaucoma is mainly concluded from experimental studies using rodent visually guided eye growth models [6–8]. Steinhart et al. found an induced mutation in collagen 8α2 that developed larger eyes and altered biomechanical behavior from inflation testing in mice [9]. Furthermore, CD1 mice also showed longer eyes, greater scleral strain in some directions at baseline, and generalized scleral thinning after glaucoma than B6 mice as two chronic experimental glaucoma models by using polystyrene bead injection into the anterior chamber [10].

In this study, we aimed to determine the shape of the central sclera quantitatively in a rat model with circumlimbal suture (CS) to compare with sclerosant injection (SI), which was previously proven to be a model of chronic ocular hypertension (OHT), and how it is affected by OHT and other clinical/ocular parameters.

## Methods

### Animal

Sixty-six adult Sprague-Dawley (SD) rats (female; 8 weeks old; 200–250 g) purchased from Animal Laboratory Supplies (Xiangya School of Medicine, Central South University, Changsha, Hunan, China) were used in this study (n = 22 rats per group). All animals were housed in comfortable conditions under a 12-h light/12-h dark cycle at 23 ± 2 °C, humidity level of 60%–70%, with free access to food and water. All surgical procedures were performed under general anesthesia using a solution of 2% sodium pentobarbital (80 mg/kg) administered by intraperitoneal injection. Oxybuprocaine hydrochloride (0.4% Oxybuprocaine; Santen, Japan) was applied as a topical anesthetic and 0.5% levofloxacin hydrochloride (Santen, Japan) was applied to prevent postsurgical infection. Animals under general anesthesia were placed on a heating blanket until they woke up.

### Experimental design

In all rats, both eyes were used as experimental specimens and untreated controls. The intraocular pressure (IOP) was measured in both awake animals’ eyes using a rebound tonometer (Tonolab, Icare, Vantaa, Finland) without topical anesthesia, on three consecutive days to establish a baseline by the same inspectors at the same time of the day (between 8 a.m. and 10 a.m.) and under similar lighting conditions. The IOP readings were taken 2 min after the surgery under general anesthesia. For the 1st week, IOP was measured daily, and for the rest of the experimental period, IOP was measured three times per week. AL and refraction recordings were taken in both eyes 3 days before the operation and at 1, 2, 4, and 24 weeks after the operation. The flash visual evoked potential (fVEP), retrograde tracing, Western blot, MASSON staining, and transmission electron microscope (TEM) were also recorded simultaneously from both eyes at 4 weeks post-surgery. Non-invasive in vivo assessment of eye structures was performed using the optical coherence tomography (OCT) system, and magnetic resonance imaging (MRI) at 4 weeks. After the AL, IOP, and refraction recordings, the first batch of rats was euthanized at 1 and 2 weeks (n = 6). Furthermore, after fVEP, MRI, and OCT measurements were taken, the second batch of rats was euthanized at 4 weeks (n = 13) and the third group at 24 weeks (n = 3) for Western blot analysis.

### Surgical technique

Procedures for the surgical technique have been described in detail previously [11, 12]. Briefly, a glass microneedle (33G, Hamilton) was positioned parallel to the vessel axis, inserted into the vessel lumen and a volume of 50 µL of lauromacrogol (Polyoxyethylene lauryl ether 1%; Tianyu, Shanxi, China) was dissolved in distilled water and slowly injected into the superior scleral vein of one eye during the SI induction. During the CS induction, a CS (7-0 nylon) was placed around the equator of the eye at approximately 1.5 mm behind the limbus (Additional file 2: Fig. S1). The suture was anchored by placing it below the conjunctiva, avoiding the major episcleral drainage veins evenly spaced around the globe at five to six anchor points. The congestion of the vortex veins could be prevented by the anterior position of the suture. The eyes of the experimental animals were treated with antibiotic eye drops after the operation. The control group was accepted without any disposal. Both eyes were performed with the same treatment in experimental groups.

### Axial length and refraction measurement

AL was measured by an ophthalmic A-scan ultrasonic diagnostic instrument (ODM-2100S, Maida, Tianjin, China). The ultrasonic probe was used to touch the corneal surface gently (frequency = 10 Hz and resolution = 0.01 mm). Then, the pupils were dilated with the instillation of eye drops containing a mixture of 0.5% tropicamide and 0.5% phenylephrine hydrochloride (Santen, Japan). Refractions were measured by streak retinoscope (YZ24B; 66 Vison Tech, Suzhou, China). The average AL and refraction were calculated and recorded.

### Magnetic resonance imaging

Details of the MRI protocol have been published previously [13]. The MRI scans (7.0T BioSpec 70/20 USR; Bruker, Billerica, MA, USA) were applied to measure AL and access the shape of the eyeball with a three-dimensional (3D) MRI. According to the positioning image, T1 and T2 imaging scanning parameters included the acquisition sequence [T1 = multislice-multiecho (MSME) and T2 = rapid acquisition with relaxation enhancement (RARE)], the thickness of acquisition layer (T1 = T2 = 0.6 mm), relaxation time (T1 = 10,188.3320 ms and T2 = 5344.0875 ms), and time to echo (T1 = 14 ms and T2 = 36 ms). The 3D imaging was performed by using commercially available software (OsiriX MD; Food and Drug Administration cleared, Pixmeo, Geneva, Switzerland).

### Flash visual evoked potential

The fVEP was performed using the device (GT-2008V-VI; GOTEC, Chongqing, China) for functional evaluation of retinas (the stimuli intensity = 10.0 cd·s/m^2^, the flash frequency = 1 Hz, and the number of flashes = 64 times). After dark adaptation for 12 h, pupils were dilated with the instillation of eye drops, the fully anesthetized animals were then placed on the examination table, and the fVEP of the unilateral eye was examined. The average value after 63 flashes was taken to calculate the P2 wave.

### Optical coherence tomography

Following the fVEP recording, the retinal structure was imaged using OCT (Micron IV, Pleasanton, CA, USA) as previously reported [14]. The ganglion cell complex (GCC) included the retinal nerve fiber layer (RNFL), ganglion cell layer (GCL), and inner plexiform layer (IPL) [15]. In brief, the corneal surface was protected using a 0.9% saline solution before the inspection. OCT images were obtained from a position located horizontally at one disc diameter superior to the optic disc. Meanwhile, ocular fundus monitoring was performed when the rats were fully anesthetized. The acquired retinal spectral domain OCT images were quantitatively analyzed using Image J v.1.8.0 after segmentation of each sublayer. During all experimental procedures, the physical condition of the rats was frequently monitored by inspection and gentle palpation.

### Retrograde tracing

To observe the survival of retinal ganglion cells (RGCs) in OHT eyes, retrograde tracing was performed as described previously with slight modifications [16]. Briefly, a surgical blade was used to make an incision to expose the bregma and posterior fontanelle on anesthetized rats. The localizations of the bilateral SC are 6 mm posterior to the bregma and 1.8 mm lateral to the midline. Two small holes (2.5 mm in diameter) were made using a mini drill on both sides of the midline. A syringe (5 μL, Hamilton, USA) was vertically inserted to a depth of 4 mm and used to slowly inject 0.5 μL of tracer (4% fluorogold dissolved in 0.9% saline, USA) into the ambilateral SC. Placement of the syringe in the target zone and tracer injection took 10 and 3 min, respectively. Following the completion of the two-site injection, 4-0 surgical threads (Ethicon, Inc., Somerville, USA) were used to suture the incision. During the whole procedure, a heating lamp was used to keep the rats warm. The mean density of retinal ganglion cells (MD-RGCs) was performed using Image J v.1.8.0 with a semi-automated method [17].

### Tissue preparation and staining

The rats were euthanized, and the eyeballs were enucleated instantly and immersed in 4% paraformaldehyde for 2 h at room temperature, and then embedded in paraffin. The paraffin sections of 4 μm was cut through the papillary optic nerve plane and stained with hematoxylin-eosin (H & E) and Masson. In Masson-stained images, collagen was dyed blue, the blue area of the posterior segment (except cornea) of the eyeball wall across ONH was taken as the numerator and the total staining area of that eyeball wall was taken as the denominator. Thus, the collagen volume fraction of the posterior segment was calculated by the percentage of the ratio between the numerator and denominator [18].

### Western blot analysis

According to the previous regions of the globe, the sclera within 4 mm outside the corneal limbus and the sclera within 4 mm from the optic nerve was defined as the peripheral sclera and the posterior sclera, respectively, by a vernier caliper (1–150 mm; SPIFFFLYER, Germany) with a resolution of 0.01 mm. The position of the peripheral sclera corresponded to the equatorial part and the central one corresponded to the PPS according to the division of the eyeball wall [1, 19]. The minced tissue samples were ground using a grinder (KZ-II, Servicebio, Wuhan, China) set at the power setting model (frequency = 60 Hz and time = 60 s). Sample protein concentration was determined by bicinchoninic acid (BCA) protein quantitative detection kit. For Western blotting, 5× sodium dodecyl sulfate (SDS) loading buffer was added to each sample, and samples were placed in a boiling water bath for 5 min before being loaded into wells of 10% sodium dodecyl sulfate polyacrylamide gel electrophoresis (SDS-PAGE) gel. After electrophoresis, proteins were transferred onto a polyvinylidene fluoride membrane. After blocking with 5% bovine serum albumin, the proteins on the membranes were immunoblotted overnight at 4 °C with the anti-alpha smooth muscle actin (αSMA, 1:1000; #19245; Cell Signaling Technology, USA) antibody. After three washes with tris buffered saline with Tween 20 (TBST), the membranes were incubated with the appropriate horseradish peroxidase-conjugated secondary antibody (1:5000; Jackson ImmunoResearch) at room temperature for 1 h. Western blot bands were detected using an enhanced chemiluminescence solution (Millipore, Bedford, MA). Densitometric analysis was performed using Image J v.1.8.0 software.

#### Transmission electron microscopy

The peripheral sclera and retina regions were located as previously mentioned [1]. Histopathological tissue preparation followed the measurement of the external diameter of eyeballs in vitro by the vernier caliper. For analysis, the globe was divided into peripheral sclera corresponding to the equatorial part and the central one corresponding to the PPS. The nasal and temporal regions were combined to focus on the anterior patterns. Tissue (diameter = 1 mm) harvested from animals was fixed by electron microscope fixative (2.5% Glutaric dialdehyde solution, Servicebio, China). The prepared niosomal formulations were characterized for their shape using TEM (HT770, HITACHI, Japan) at 80 kV. A TEM micrograph was taken at suitable magnification after staining.

### Statistical analysis

The data are presented as mean ± standard deviation of results from at least three independent experiments [the data are presented as mean ± standard error of the mean (SEM) in Table 1 and Additional file 1: Table S1]. The statistical significance of the experimental differences between groups was analyzed using the Student’s t-test, and comparisons among groups were analyzed using one-way analysis of variance (ANOVA). Statistical analyses were performed on GraphPad Prism 8.0 (GraphPad Software, La Jolla, CA, USA).

**Table 1:** Intraocular pressure measurements at different time points in each group (mmHg)

Experimental periods	Con	SI	CS
3 days preop	11.0 ± 1.2	12.1 ± 1.4	10.9 ± 1.3
2 min postop	12.2 ± 1.5	17.5 ± 9.0	61.4 ± 10.4**
1 day postop	11.9 ± 1.1	17.9 ± 3.2*	25.1 ± 13.6**
2 days postop	11.3 ± 0.8	16.7 ± 1.5**	32.1 ± 12.2**
3 days postop	12.0 ± 0.7	17.8 ± 1.6**	31.2 ± 4.9**
7 days postop	12.6 ± 1.3	23.2 ± 3.5**	32.9 ± 8.3**
14 days postop	11.4 ± 0.7	23.1 ± 3.7**	31.5 ± 5.8**
21 days postop	12.7 ± 0.8	23.0 ± 4.0**	32.3 ± 5.9**
28 days postop	13.0 ± 1.2	19.7 ± 3.9**	28.1 ± 5.5**
56 days postop	11.9 ± 1.4	14.1 ± 2.1*	25.6 ± 3.3**
168 days postop	11.9 ± 1.9	12.9 ± 2.1	13.0 ± 2.6

*Con* = control group; *CS* = circumlimbal suture group; *SI* = sclerosant injection group; *preop* = preoperative; *postop* = postoperative

*Data are presented as mean ± SEM; *P < 0.05, **P < 0.01, paired Student’s t-test

## Results

### Elongation of AL and reduction of RGCs after IOP elevation induced by the CS

**Fig. 1:**
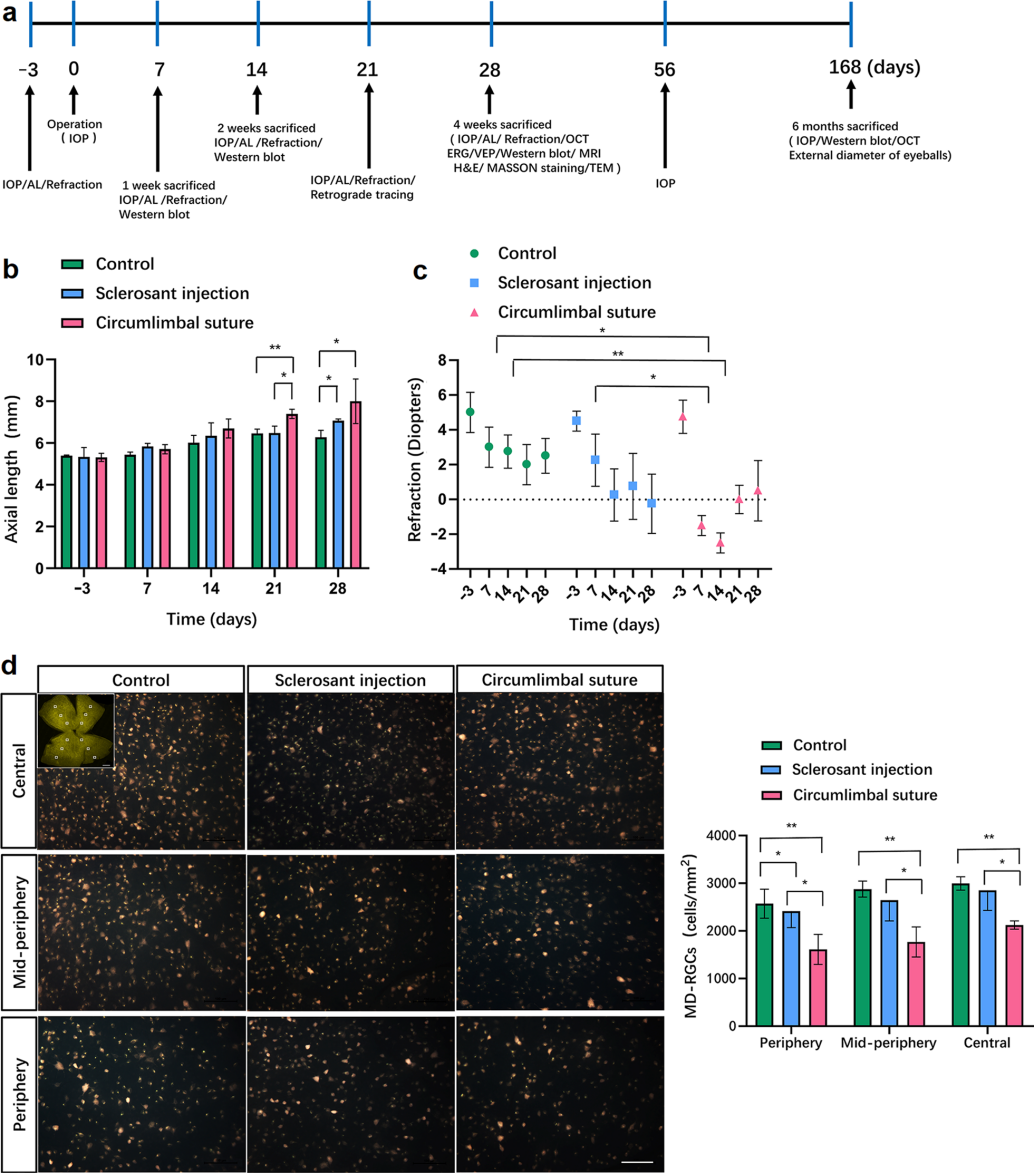
Study scheme, results of axial length (AL), and retinal ganglion cells (RGCs) in ocular hypertension eyes. **a** Schematic of the experimental time points; **b** Verifying elongated AL measured by ultrasound in circumlimbal suture (CS) models at 3 days before the operation, 7, 14, 21, and 28 days after the operation (n = 6, **P < 0.01 compared with the control group, **P < 0.01 compared with sclerosant injection (SI) group at 21 and 28 days after the operation). **c** Verifying changes of diopters in CS eyes at 3 days before the operation, 7, 14, 21, and 28 days around the perioperative period (n = 6, *P < 0.05 compared with the control group at 7 and 14 days after the operation, *P < 0.05 compared with SI group at 7 days after the operation); **d** The mean density of RGCs (MD-RGCs) retrogradely labeled by fluorogold after 28 days of operation in the CS rats (n = 6, **P < 0.01 compared with the control group in the peripheral, mid-peripheral and central retina, *P < 0.05 compared with SI group in the peripheral, mid-peripheral and central retina). Scale bar = 50 μm

To compare the differences in AL before and after the operation, the IOP of 66 rats was measured by a contact tonometer (Tonolab), AL was measured by ultrasound, and the refraction was examined by a retinoscopy (Fig. 1a). To avoid cross-reaction caused by the intravenous injection of angiosclerotic agent, both eyes were treated consistently in three groups. The elevation of IOP was observed in eyes of the rats from the experimental groups for more than 2 months. More specifically, there was a gradual increase in IOP from a basal value of 12.6 ± 1.3 to 31.2 ± 4.9 mmHg in the CS group 3 days after the operation; the corresponding increase in the SI group was 17.8 ± 1.6 mmHg. The average IOP in the CS group over the 2-month experimental period was 25.6 ± 3.3 mmHg; the SI group showed an IOP value of 14.1 ± 2.1 mmHg. The control group maintained an IOP value of 11.9 ± 1.4 mmHg throughout the experiment (Table 1). The increased ALs of the eyes were observed during the experimental period in three groups; the ALs in the CS group were significantly increased 3 weeks after the operation (Fig. 1b). Additionally, the decreased diopters of the CS group were observed after 1 week of operation compared to the control and SI groups, which were significantly decreased after 2 weeks of operation (Fig. 1c).

After confirming IOP elevation, RGC survival and retinal stress were evaluated by fluorogold retrograde tracing assay after 4 weeks of operation. The decreased MD-RGCs were revealed significantly in the peripheral retina in OHT models. The CS group showed a reduction of MD-RGCs in the mid-peripheral and central retina (Fig. 1d).

### Morphological and visual functional changes in different regions of OHT eyes with elongated AL

**Fig. 2:**
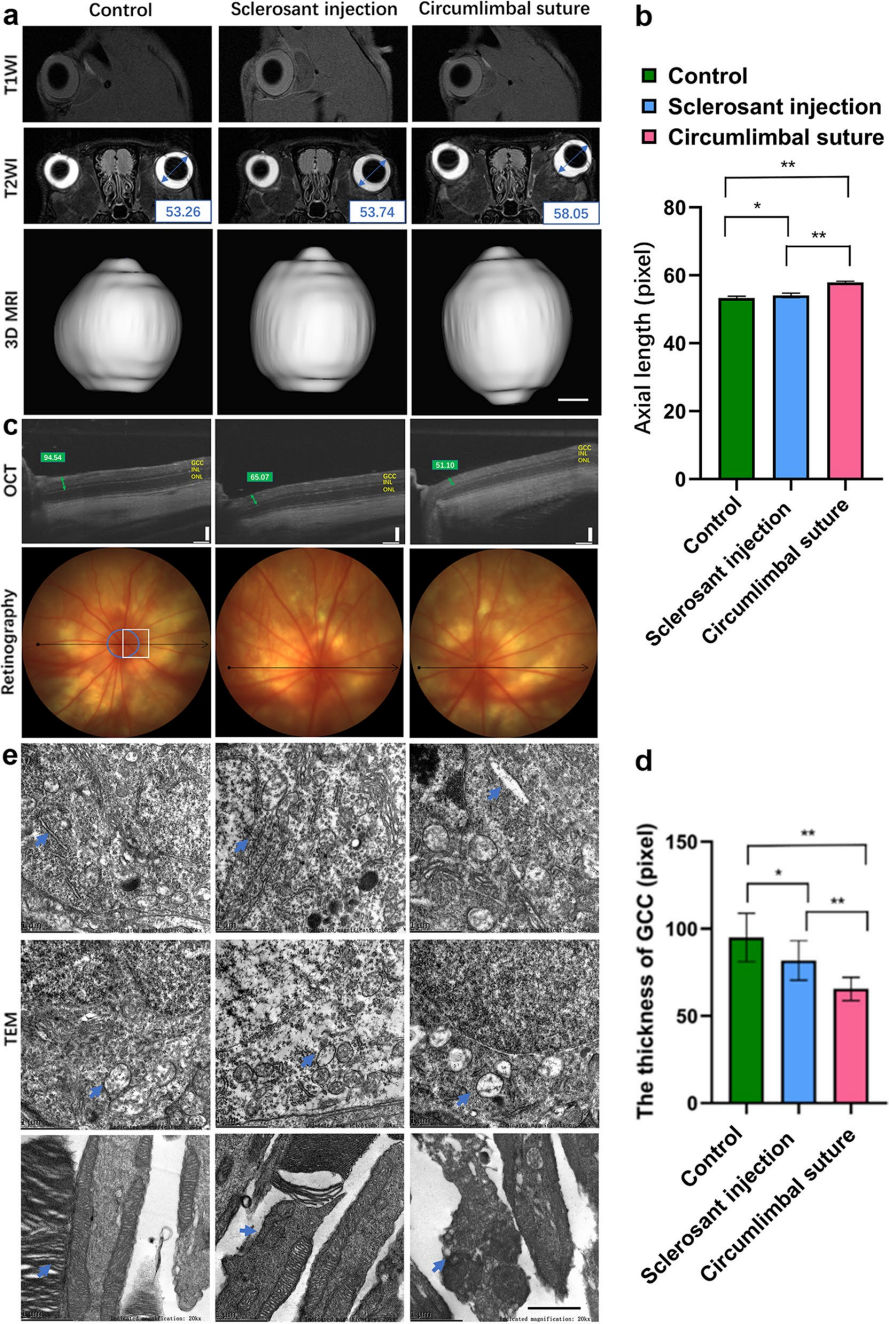
Morphological changes of different regions of eyeballs. **a** Typical T1-weighted images (TIWI), typical T2-weighted images (T2WI), and representative 3-dimensional (3D) MRI scan in all three groups (n = 4). Scar bar = 1 mm. The right eye of six adult rats after 28 days of operation. The measured data of axial length (AL) by MRI was 53.26, 53.74, and 58.05 pixels in the control, sclerosant injection (SI), and circumlimbal suture (CS) groups, respectively; **b** Comparison of AL by MRI in CS group (n = 4, **P < 0.01 compared with the control group, **P < 0.01 compared with SI group); **c** Rat retinal imaging by optical coherence tomography (OCT) across the center of the optic nerve head (ONH). The measured data of the ganglion cell complex (GCC) was 94.54, 65.07, and 51.10 pixels in the control, SI, and CS groups after 28 days of operation, respectively; **d** Comparison of the thickness of GCC in the CS group (n = 6, **P < 0.01 compared with the control group, **P < 0.01 compared with SI group). Scale bar = 100 μm; **e** Representative images of organelles in retinal ganglion cells (RGCs) and photoreceptor cells in the peripheral retina of ocular hypertension (OHT) models. Endoplasmic reticulum (ER) and mitochondria in RGCs were observed by transmission electron microscope (TEM) after 28 days of operation (arrows)

To further study the shape changes of increased AL after early IOP elevation induced by the CS compared with the SI, the AL was measured by an MRI scan after 28 days of operation. The CS group showed a longer AL than the SI and control groups (Fig. 2a, b). The thinning of the GCC around the ONH in the OHT rats was observed by the OCT 28 days after the operation; the GCC was thinner around the ONH in the CS group than in the SI group. Meanwhile, the GCC thickness decreased in the SI group compared with the control group (Fig. 2c, d).

Nine retinal images photographed at 5/6 of the retinal radius (3.68 mm from the optic disc) was randomly selected from each group to observe the morphological organelles in RGCs and photoreceptor cells in all three groups. The CS group showed a significant swelling of the RGC body, an expansion of the endoplasmic reticulum (ER), an enlargement of mitochondrial volume, a reduction of membrane density, rupture, or disappearance of mitochondrial cristae; the inner and outer segments of the photoreceptor cells were either partly broken or missing (Fig. 2e).

The thicknesses of the peripheral, mid-peripheral, and central GCC were measured by H & E staining (Additional file 3: Fig. S2). The decreased thickness of peripheral and central GCC was observed in the CS group (*P < 0.05 compared with the control or SI group, Additional file 1: Table S1). The anterior segment and the optic nerve were observed by H & E staining. The CS group presented a deformed peripheral retina, extensive degeneration, and vacuolation of the optic nerve (Additional file 4: Fig. S3).

Furthermore, the analysis demonstrated a positive correlation between IOP and AL (R^2^ = 0.3785, *P* = 0.0066), and a negative correlation between IOP and the thickness of GCC (R^2^ = 0.6521, *P* < 0.001) (Additional file 5: Fig. S4).

The fVEP showed dysfunction of nerve conduction with visual stimulus in the OHT groups, which was more significant in the CS group (Additional file 6: Fig. S5).

### Collagen volume fraction and collagen crimp in different regions of OHT eyes with elongated AL

**Fig. 3:**
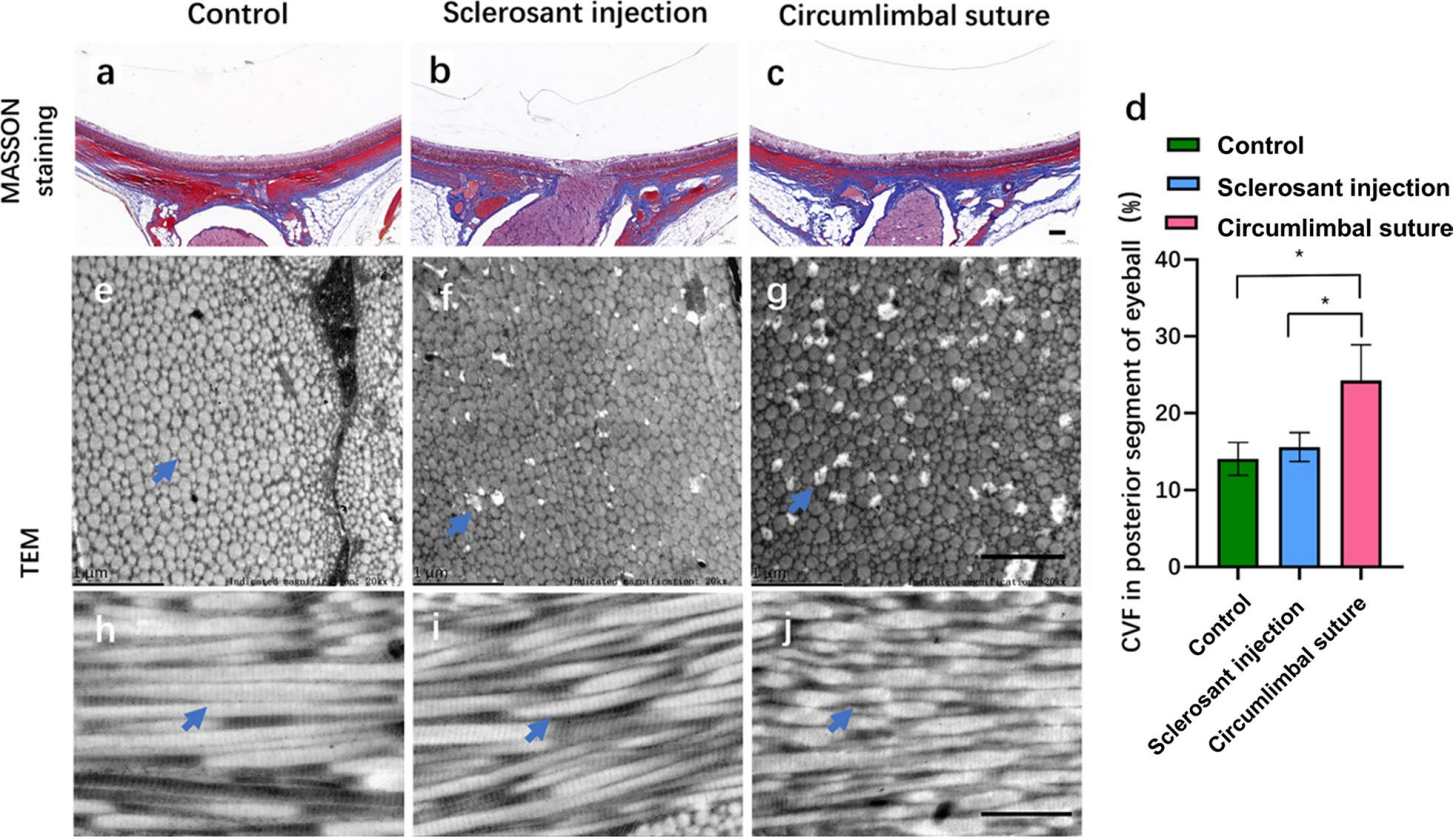
The collagen volume fraction (CVF) of posterior eyeball wall and collagen crimp of peripheral sclera. **a**–**c** The CVF was measured by MASSON staining after 28 days of operation in circumlimbal suture (CS) group. Scale bar = 100 μm; **d** Comparison of the CVF in posterior segment of eyeball wall in CS group [n = 6, *P < 0.05 compared with sclerosant injection (SI) group, *P < 0.05 compared with control group]; **e**–**g** Representative transmission electron microscope (TEM) images of the outer peripheral sclera after 28 days of operation, showing lamellar structure formed by collagen fibril bundles in transverse (Tc) section (n = 4). The peripheral scleral collagen was dissolved and absent in the CS group (arrows). Scale bar = 1 μm; **h**–**j** Representative TEM images of the outer peripheral sclera after 28 days of operation, showing lamellar structure formed by collagen fibril bundles in longitudinal (Lc) sections (n = 4). The peripheral scleral collagen crimp was found in the CS group (arrows). Scale bar = 1 μm

Collagen volume fraction, the ratio of collagen fibers in the posterior segment of the eyeball based on Masson staining was calculated, across the optic nerve showed an increased percentage in the CS group. Additionally, collagen fiber in the peripheral sclera was dissolved and absent in the CS group under an electron microscope; the peripheral scleral collagen crimp was found in the CS group (Fig. 3).

### The expression of αSMA in different regions of OHT eyes with elongated AL

**Fig. 4:**
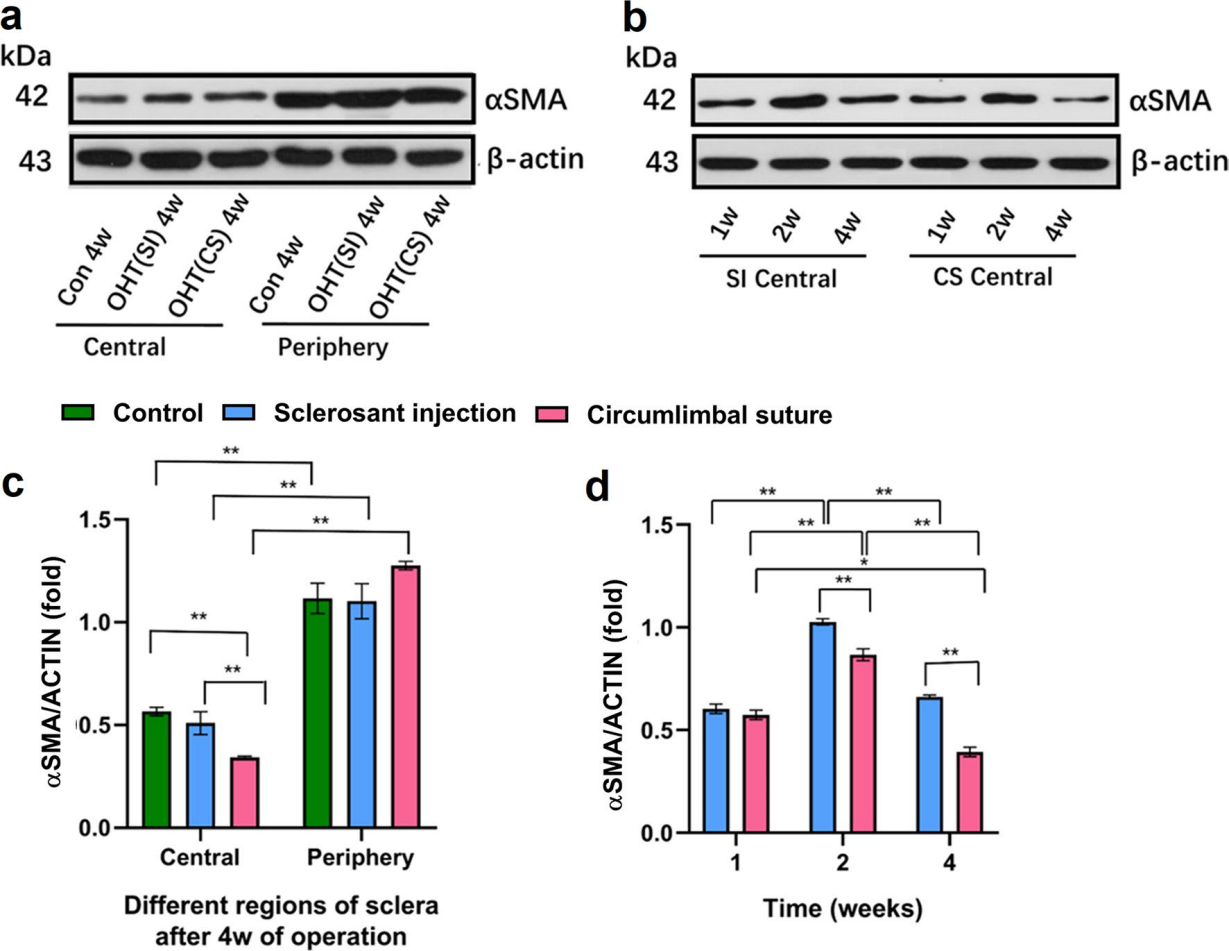
Representative Western blots, quantification of αSMA in different periods and regions of scleras in models. **a** Representative Western blots of αSMA in central and periphery scleras 4 weeks postoperatively in three groups; **b** Representative Western blots of αSMA 1, 2, 4 weeks postoperatively in two experimental groups; **c** Expression of αSMA in central and periphery scleras 4 weeks post-operatively in circumlimbal suture (CS) group [n = 6, **P < 0.01 compared with control or sclerosant injection (SI) group in central scleras]; **d** Expression of αSMA 1, 2, 4 weeks post operatively in CS group (n = 6, **P < 0.01 compared with SI group after 4 weeks of operation, **P < 0.01 in CS group after 4 weeks of operation compared with 2 weeks). The central sclera corresponded to the posterior sclera according to the division of the eyeball wall [19]

To evaluate the structural molecular changes involved in the different regions and periods after IOP elevation, Western blot analysis was performed after the operation for αSMA, an actin isoform that is expressed predominantly in vascular smooth muscle cells and plays an important role in fibrogenesis. A significantly increased expression of αSMA was noted in the peripheral sclera compared to the central sclera after 4 weeks of operation in three models. However, the central sclera showed lower expression of the protein in the CS group than in the control and SI group after 4 weeks of operation. Additionally, Western blot analysis was used to determine the peak expression of αSMA in the 4 weeks after operation; the central sclera showed higher expression of αSMA in the CS group than that in the SI group 2 weeks after the operation (Fig. 4).

## Discussion

Risk factors for POAG—the most common form of glaucoma—include older age, elevated IOP, sub-Saharan African ethnic origin, positive family history, and high myopia [20]. In a prospective observational cohort study where researchers analyzed 245 eyes of 135 glaucoma patients followed up for a mean duration of 6.08 years, both RNFL and visual field progression rates were faster in glaucomatous myopic eyes with an AL ≥ 26.5 mm than in eyes with an AL < 26.5 mm [21]. This study demonstrated the presence of regional differences in the sclera and extension of the ocular axis in rat eyes with elevated IOP in CS and SI groups as OHT models [11, 12]. Scleral structural changes are related to various factors, such as aging, disease, extracellular matrix (ECM) structure, biomechanics, and scleral response to IOP [1]. The change of PPS with age could have an impact on the overall biomechanical environment of ONH in the elderly [5]. Moreover, changes to the scleral microstructure may be caused by the response of the sclera to acute elevated IOP and suture-related stimulus, previously mentioned in the CS group (maximum IOP = 61.4 ± 10.4 mmHg), but not in the SI model (maximum IOP = 23.0 ± 4.0 mmHg).

In the SI model, the IOP increased from 4 to 5 weeks after surgery, while in the CS model, the IOP increased at the 4th week and reached a stable stage. Under the guidance of non-invasive A-mode ultrasound, we found that the ocular AL was changed from 3 to 4 weeks, so the 4th week was used as the main observation period of this study. Francisconi et al. demonstrated a positive correlation between AL and average macular ganglion cell-inner plexiform layer measured by spectral-domain OCT [4]. Our finding of positive correction for extension of the ocular axis was applied, which is in line with previous findings [22]. Local deformation was determined between the optic nerve papilla and the peripheral sclera with elevated IOP [23]. Wang et al. described a morphological change in a chronic rabbit OHT model which was induced by limbal buckling and described a non-spherical morphological change of eyeball after suturing. However, the effect of IOP-related extension of the ocular axis was not reported [22].

**Fig. 5:**
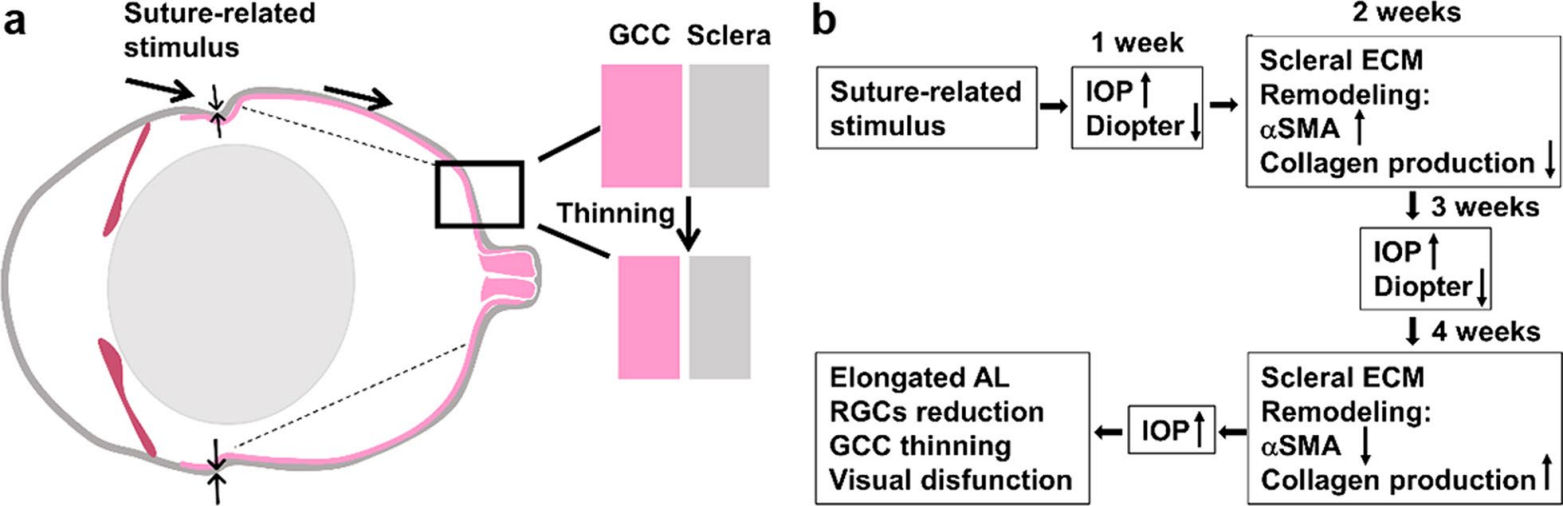
Paradigm for the central scleral change involved in circumlimbal suture associated eyes with ocular hypertension. **a** The ganglion cell complex (GCC) thins followed by scleral thinning that results from extracellular matrix (ECM) remodeling during suture-related stimulus; **b** These suture-related pressure signals increase the fibroblasts’ response to increase αSMA expression and decrease collagen production after 2 weeks of the operation. Central scleral ECM remodeling with elevated intraocular pressure (IOP) from suture-related stress after 2 weeks of the operation, leading to increased collagen production and decreased expression of αSMA

The thickness of GCC, a complex layer composed of retinal plexus layer, ganglion cell layer, and RNFL, decreased in the OHT models after operation compared with the control group. The CS group showed a reduced thickness of GCC in the central retina compared with SI, and the outcome of H & E staining showed a similar outcome in OCT. Meanwhile, the CS group showed a significantly deformed eyeball wall under mechanical pressure in the peripheral retina and extensive degeneration and vacuolation of the optic nerve. In a previous study, posterior bowing of PPS and the scleral canal were observed in humans [24] and other animal eyeballs, but not rat eyeballs [23, 25]. The non-linear effect was probably associated with the deformation-induced stiffening resulting from inhomogeneously crimped collagen fibers [26]. The pressure conduction from the peripheral sclera to the central position likely reduced the transport of oxygen and nutrients and may be even acted on the central RGCs. Additionally, lasting suture stimulation may be related to the deformation of collagen fibers and the increase of fibrogenic cell activity in elongated AL in CS models (Fig. 5).

The CS with suture compression of peripheral sclera vessels and the SI with sclerosing agent blocking episcleral vein were considered as the methods of pressure-dependent OHT model [8, 11, 27, 28], concomitant with reductions in positive scotopic threshold response, RNFL thickness, RGC density, and photoreceptor function [29, 30]. Zhao et al. confirmed that the function of RGCs was reversed after 8 weeks of suture removal in the mouse CS model, but that it was irreversible after 12 weeks because the capacity of RGCs to functionally recover from chronic IOP elevation was dependent on the duration of IOP elevation [31]. In our rat OHT models, the thinning peripapillary GCC, extensive degeneration, and vacuolation of ONH are likely involved in the decrease of visual function in the CS group compared with the SI group. Boote et al. suggested that fibroblasts control scleral remodeling and alter tissue-level biomechanics in response to a signaling cascade from the retina to the sclera that is ultimately stimulated by vision [1]. We hypothesize that the decreased αSMA expression in the central sclera may be related to the dissolution and loss of collagen fibers which affect the surrounding environment of fibroblasts in the CS group after 2 weeks of operation. Interestingly, Szeto et al. investigated the cellular architecture of normal human PPS cryosections from non-glaucomatous eyes and reported that fibrillar actin and αSMA differed in cellular density and nuclear morphology of PPS fibroblasts [32].

In the deformation response of the lamina cribrosa and sclera of human donors, it was found that sclera deformation was different in the lamina cribrosa and retinal ganglion axons [33]. There are few animal models of research on the change of the shape in POAG with the inappropriate extension of the ocular axis. Tatewaki et al. found that the ocular morphology is a near-spherical shape in glaucomatous eyes or glaucomatous myopic eyes, while the shape in myopic eyes is similar to an ellipsoid based on MRI analysis [34]. Here, the morphological changes observed in rat eyeballs were similar to those seen in a chronic rabbit OHT model, which was induced by limbal buckling and described the non-spherical morphological change of eyeballs after suturing [22]. Pijanka et al. used wide-angle X-ray scattering (WAXS) and reported that spatial dynamic changes in collagen fibril anisotropy occur in the posterior sclera of CD1 mice with bead-induced chronic IOP elevation and axonal damage changes [35]. Meanwhile, Cone-Kimball et al. emphasized the importance of the dynamic response of the sclera to OHT mice through ultrastructural investigations [36].

CS was described to be less invasive with better maintenance of clear optical media compared to episcleral vein cauterization or sclerosis of the outflow pathway; the IOP can also be titrated via the suture tightness [27]. However, this technique can lead to IOP spikes which could give rise to retinal ischemia and ocular complications like cataracts or hyphema [8, 11, 12, 28, 37]. To the best of our knowledge, there have been only a few studies describing the influence of regional differences of the sclera in the ocular hypertensive animal model. This study demonstrated a model that was constructed by the CS method and analyzed the characteristics of the model from the elevation of IOP, loss of RGCs, reduction of visual function, and changes in the sclera, especially collagen deformation, providing an alternative model in sclera stiffening and biomechanical studies around the optic nerve papilla.

Our study has some limitations. The peak of IOP (61.4 ± 10.4 mmHg) in the CS group at 2 min after surgery was equivalent to acute IOP elevation but not the chronic OHT model as the SI course. The mean IOP level, IOP fluctuation, and peak IOP were closely associated with incident human glaucoma and its progressive worsening [10]. The transient elevated IOP had an impact on the central regional RGCs, even on the photoreceptor cells based on our additional electroretinogram (ERG) inspection results. In previous studies, the duration of elevated IOP was 16 weeks in a CS model [31], and 24 weeks in an SI model [12]. In both models, the IOP returned to the preoperative level at the 6th month. Therefore, the analysis of αSMA after 4 weeks of operation was only for the clinical processes from acute IOP elevation to the chronic course, and the expression of αSMA in the clinical course of OHT could not be studied. The decrease in refraction of control groups relative to the pre-surgical baseline may be limited by the number of animals. The decrease after 1 week for the CS group may have happened instantaneously after ligation from the change in eye shape and not OHT per se. Therefore, it is necessary to expand the sample size to determine the change of refraction affected by AL and age.

Our research needs further investigations, such as the analysis of other proteins, like matrix metalloproteinase, hypoxia-inducible factor, and fibrillar actin that might be involved in the ocular changes [38], and a more in-depth discussion of the relationship between other OHT models and ocular axis concerning simulating the development of glaucoma, including changes to fibroblasts, proteoglycans, and other scleral composition. Scleral therapy, such as with small molecule collagen crosslinker [39], glyceraldehyde, acetone aldehyde, and genipin [40], with a focus on crosslinking to the PPS region, have been used in research on scleral stiffening therapy and can have a beneficial effect by reducing the magnitude of biomechanical strains within the lamina cribrosa [41] in glaucoma and myopia. Given the increasing burden of scleral-related visual impairment in the world, there are still only limited animal models to study the mechanism of high IOP on the sclera in vivo. With the further validation of this model, we hope to help answer controversial problems in the future, such as whether sclera sclerosis or sclera hardening around the ciliary body can protect the development of glaucoma. The optimal animal model for scleral-related eye disorders may provide a reference for further treatment.

## Conclusions

Here, we demonstrated that elongated AL and different regional scleral changes in the early stage of CS- vs. SI-induced OHT in rat models involved changes in the expression of αSMA in different regions and time points after the operation. These results support the idea that dynamic changes in scleral form and structure play a role in the development of experimental glaucoma in rats, and potentially in humans too. The initial findings regarding the capability of the CS model to induce scleral changes may aid in the development of clinical treatment and differential diagnosis in open-angle glaucoma with elongated ocular AL, such as in progressive myopia. A better understanding of scleral biomechanics in glaucoma can improve our ability to predict which leads to new therapeutic approaches.

## Supplementary Information

**Additional file 1**: **Table S1.** Comparison of morphological examination items 4 weeks after the operation in each group.

**Additional file 2**: **Figure S1.** Slit-lamp biomicroscopic photography, intraocular pressure (IOP) measurement, and schematic diagram of operation methods

**Additional file 3**: **Figure S2.** Verifying changes to the thickness of the ganglion cell complex (GCC) in ocular hypertension (OHT) models

**Additional file 4**: **Figure S3.** Hematoxylin-eosin (H & E) staining of the anterior segment and the optic nerve

**Additional file 5**: **Figure S4.** Correlation analysis of intraocular pressure (IOP), axial length (AL), and the thickness of ganglion cell complex (GCC) in the rats at 4 weeks after operation

**Additional file 6**: **Figure S5.** Results of electrophysiology in ocular hypertension (OHT) eyes

## Data Availability

Not applicable.

## Abbreviations

AL: Axial length
αSMA: Anti-alpha smooth muscle actin
CS: Circumlimbal suture
CVF: Collagen volume fraction
ECM: Extracellular matrix
ER: Endoplasmic reticulum
ERG: Electroretinogram
fVEP: Flash visual evoked potential
GCC: Ganglion cell complex
H&E: Hematoxylin-eosin
IOP: Intraocular pressure
MD-RGCs: The mean density of retinal ganglion cells
MRI: Magnetic resonance imaging
OCT: Optical coherence tomography
OHT: Ocular hypertension
ONH: Optic nerve head
POAG: Primary open-angle glaucoma
RGCs: Retinal ganglion cells
RNFL: Retinal nerve fiber layer
SI: Sclerosant injection
TEM: Transmission electron microscope
