# Supplementary material for: Regional differences of the sclera in the ocular hypertensive rat model induced by circumlimbal suture
Source: Eye Vis (Lond). 2023 Jan 4;10:2. doi: 10.1186/s40662-022-00319-w (PMC9811703; doi:10.1186/s40662-022-00319-w)
Supplement: Supplementary file 1 — Additional file 1: Table S1. Comparison of morphological examination items 4 weeks after the operation in each group. [file 40662_2022_319_MOESM1_ESM.docx]

**Table S1.** Comparison of morphological examination items 4 weeks after the operation in each group.

| **Parameters** | **Con** | **SI** | **CS** |
| --- | --- | --- | --- |
| No. of RGCs in the central retina (cells/mm^2^) | 2995.2 ± 141.5 | 2767.6 ± 201 | 2121.1 ± 87.2^**^ |
| No. of RGCs in the mid-peripheral retina (cells/mm^2^) | 2875.9 ± 168.1 | 2646.0 ± 437.1 | 1768.0 ± 315.0^**^ |
| No. of RGCs in the peripheral retina (cells/mm^2^) | 2570.0 ± 304.1 | 2214.7± 208.3^*^ | 1611.3± 315.2^**^ |
| Thicknesses of the central GCC by H & E staining (μm) | 70.8 ± 1.3 | 66.9 ± 0.3 | 65.3 ± 1.8 |
| Thicknesses of the peripheral GCC by H & E staining (pixel) | 56.6 ± 2.2 | 48.7 ± 1.6^*^ | 43.8 ± 2.2^*^ |
| CVF of the posterior segment of the eyeball wall (%) | 12.9 ± 1.8 | 16.0 ± 1.0 | 30.2 ± 3.1^*^ |
| Axial length by ultrasound (mm) | 5.8 ± 0.4 | 6.3 ± 0.2^*^ | 7.1 ± 0.4^**^ |

*No.* = numbers; *Con* = control group; *CS* = circumlimbal suture group; *CVF* = collagen volume fraction; *GCC* = ganglion cell complex; *H & E* = hematoxylin-eosin; *RGCs* = retina ganglion cells; *SI* = sclerosant injection group

^*^Values represent the mean ± SEM; ^*^*P* < 0.05, ^**^*P* < 0.01, by paired Student’s t*-*test.
