# Supplementary material for: Regional differences of the sclera in the ocular hypertensive rat model induced by circumlimbal suture
Source: Eye Vis (Lond). 2023 Jan 4;10:2. doi: 10.1186/s40662-022-00319-w (PMC9811703; doi:10.1186/s40662-022-00319-w)
Supplement: Supplementary file 2 — Additional file 2: Figure S1. Slit-lamp biomicroscopic photography, intraocular pressure (IOP) measurement, and schematic diagram of operation methods [file 40662_2022_319_MOESM2_ESM.docx]

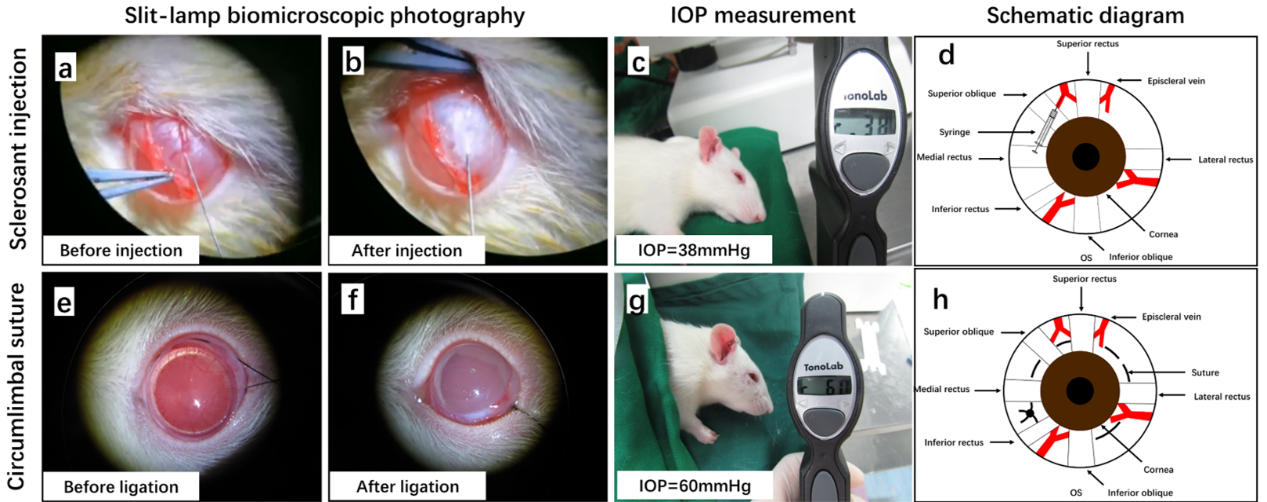
**Fig. S1** Slit-lamp biomicroscopic photography, intraocular pressure (IOP) measurement, and schematic diagram of operation methods. **a** Before the sclerosant injection (SI), superior scleral vein blood vessels were clear and full; **b** After injection of the sclerosing agent, the color of the superior scleral vein and surrounding sclera turned white; **c** The value of IOP was 38 mmHg at 2 min after the injection; **d** The schematic diagram of the method of sclerosant injection (SI) by a syringe. **e** Before suture ligation, the rat’s cornea was transparent and the limbal vascular network was well circumscribed; **f** After suture ligation, the cornea experienced edema and the limbal vascular network turned white 2 min after the ligation due to ischemia; **g** The value of IOP was 60 mmHg at 2 min after the ligation; **h** Schematic diagram of the method of circumlimbal suture (CS) by a nylon suture. Figure in the anatomy of extraocular muscles and blood vessels adapted from Shareef et al. Reproduced with permission of Elsevier [13].
