# Supplementary material for: Regional differences of the sclera in the ocular hypertensive rat model induced by circumlimbal suture
Source: Eye Vis (Lond). 2023 Jan 4;10:2. doi: 10.1186/s40662-022-00319-w (PMC9811703; doi:10.1186/s40662-022-00319-w)

**Fig. S2** Verifying changes to the thickness of the ganglion cell complex (GCC) in ocular hypertension models. A schematic diagram of measurement and positioning of eyeball GCC thickness is presented by hematoxylin-eosin (H & E)-stained sagittal sections including the center of the optic nerve. To better represent the morphological changes in the retina, 12 points of retinal thickness and GCC (a multi-layer, which corresponded exactly to the anatomical distribution of retinal ganglion cells (RGCs) in the retina) thickness per retinal slice including the optic nerve head (ONH) were measured using Image J v.1.8.0 software with the following parameters: perpendicular to the retinal pigment epithelium layer as well as ± 360, ± 720, ± 1080, ± 1440, ± 1800, and ± 2160 μm away from the center of the optic nerve. Scale bar = 500 μm.


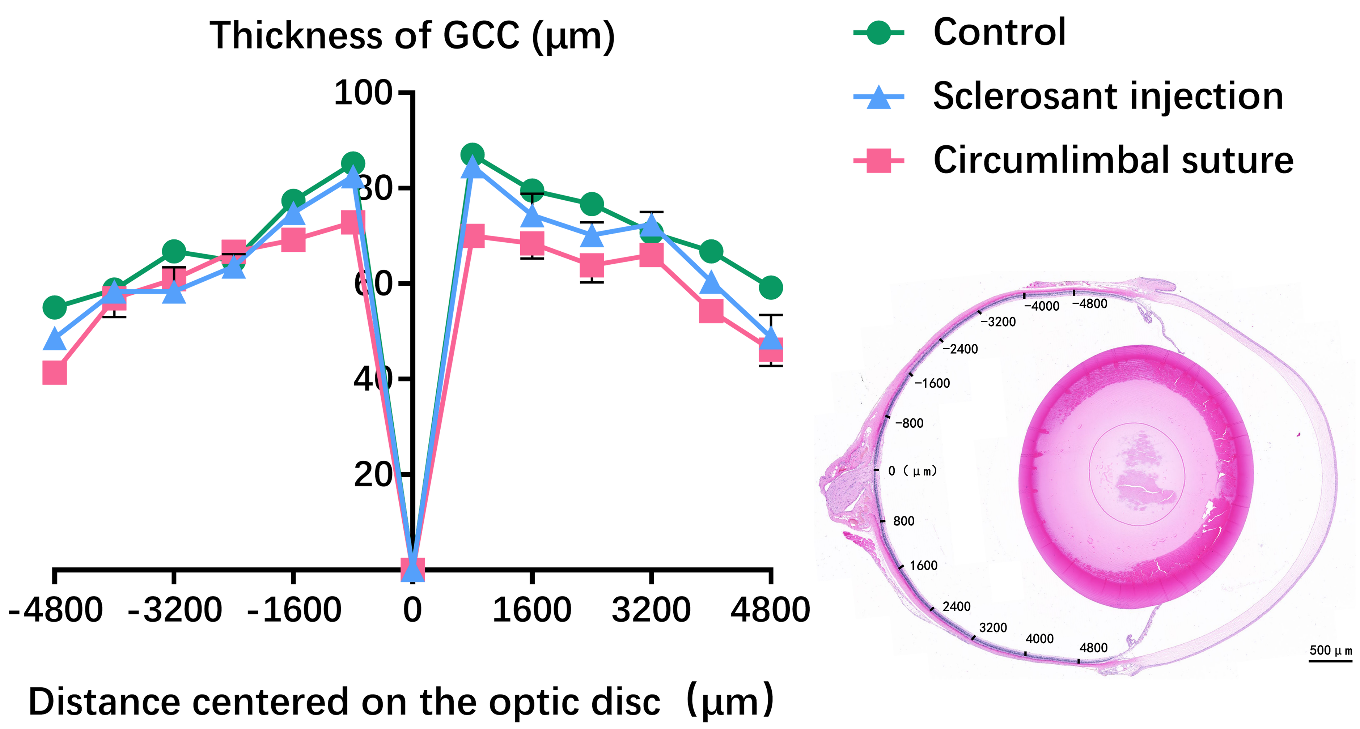

Supplement: Supplementary file 3 — Additional file 3: Figure S2. Verifying changes to the thickness of the ganglion cell complex (GCC) in ocular hypertension (OHT) models [file 40662_2022_319_MOESM3_ESM.docx]
