# Supplementary material for: Regional differences of the sclera in the ocular hypertensive rat model induced by circumlimbal suture
Source: Eye Vis (Lond). 2023 Jan 4;10:2. doi: 10.1186/s40662-022-00319-w (PMC9811703; doi:10.1186/s40662-022-00319-w)

**Fig. S3** Hematoxylin-eosin (H & E) staining of the anterior segment and the optic nerve. **a** In the control group, the limbal and subconjunctival tissues were arranged neatly; **b** In similar sites, the subconjunctival tissue structure and Tenon’s capsule (Tc) of the SI group were destroyed and the tissue gap widened; **c** The retina (R) behind the ciliary body (Cd) was compressed and deformed in the circumlimbal suture (CS) group. Scale bar = 100 μm; **d** Photomicrographs of optic nerve sections from each group stained with hematoxylin-eosin (H & E) after 28 days of operation; **e** The control group had an intact optic nerve; **f** The sclerosant injection (SI) group showed a few vacuolations of the optic nerve, and the CS group presented extensive degeneration and vacuolation of the optic nerve (arrows). Scale bar = 50 μm.


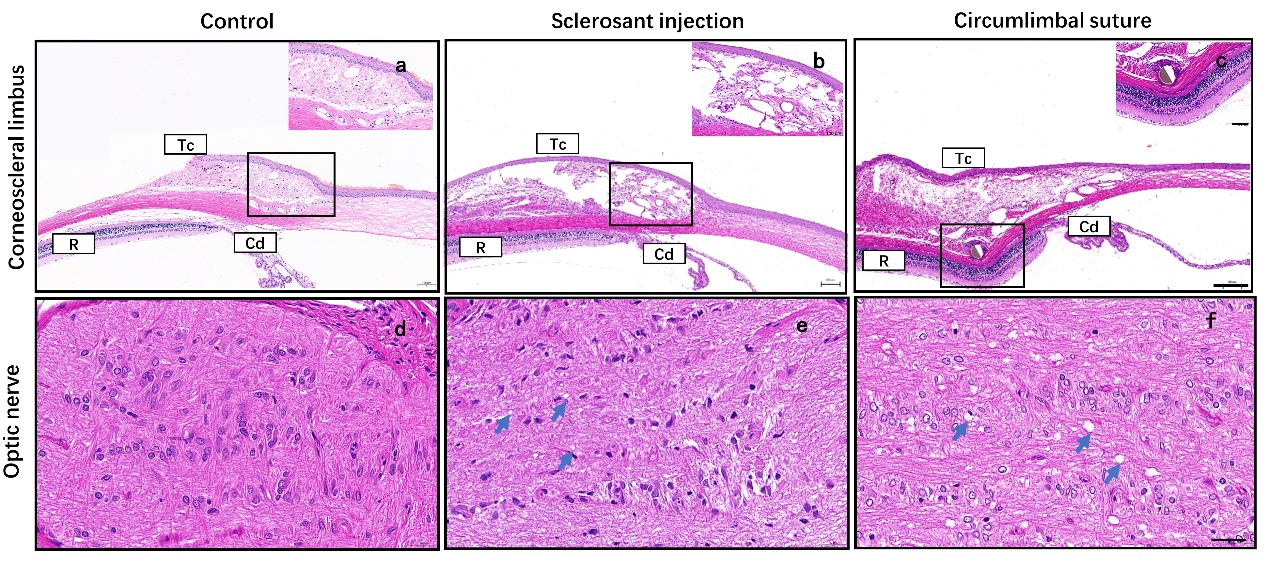

Supplement: Supplementary file 4 — Additional file 4: Figure S3. Hematoxylin-eosin (H & E) staining of the anterior segment and the optic nerve [file 40662_2022_319_MOESM4_ESM.docx]
