# Supplementary material for: Regional differences of the sclera in the ocular hypertensive rat model induced by circumlimbal suture
Source: Eye Vis (Lond). 2023 Jan 4;10:2. doi: 10.1186/s40662-022-00319-w (PMC9811703; doi:10.1186/s40662-022-00319-w)
Supplement: Supplementary file 5 — Additional file 5: Figure S4. Correlation analysis of intraocular pressure (IOP), axial length (AL), and the thickness of ganglion cell complex (GCC) in the rats at 4 weeks after operation [file 40662_2022_319_MOESM5_ESM.docx]

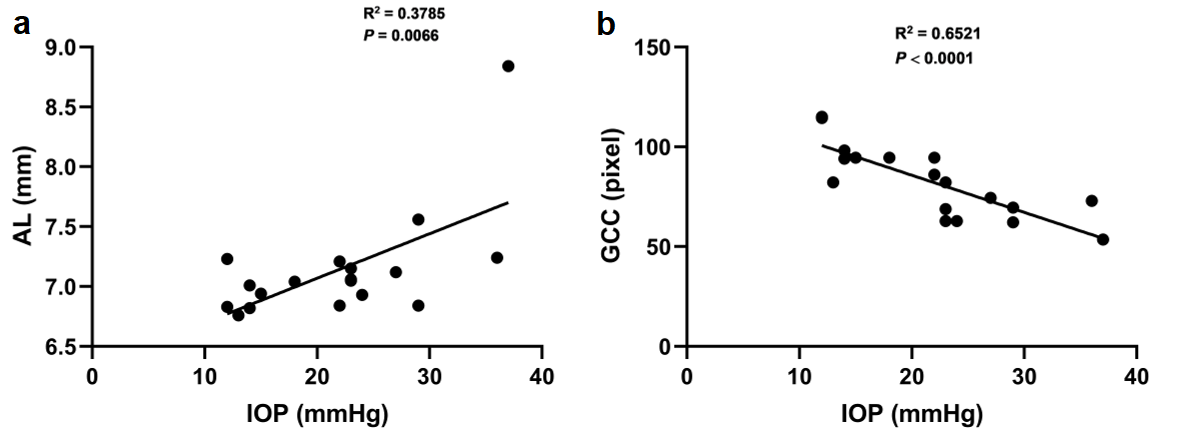
**Fig. S4** Correlation analysis in the rats at 4 weeks after the operation. **a** The correlation between intraocular pressure (IOP) and axial length (AL) (R^2^ = 0.3785, *P* = 0.0066); **b** The correlation between IOP and the thickness of ganglion cell complex (GCC) (R^2^ = 0.6521, *P* < 0.001).
