# Supplementary material for: Regional differences of the sclera in the ocular hypertensive rat model induced by circumlimbal suture
Source: Eye Vis (Lond). 2023 Jan 4;10:2. doi: 10.1186/s40662-022-00319-w (PMC9811703; doi:10.1186/s40662-022-00319-w)

**Fig. S5** Results of flash visual evoked potential (fVEP) in ocular hypertension (OHT) eyes. In the analysis for the latent period of P2-wave in fVEP examination (n = 6, **P* < 0.05 compared with control or sclerosant injection (SI) group). Measured data of latent period by fVEP was 116.5, 126.5, and 145 ms in the control, SI, and circumlimbal suture (CS) groups, respectively.


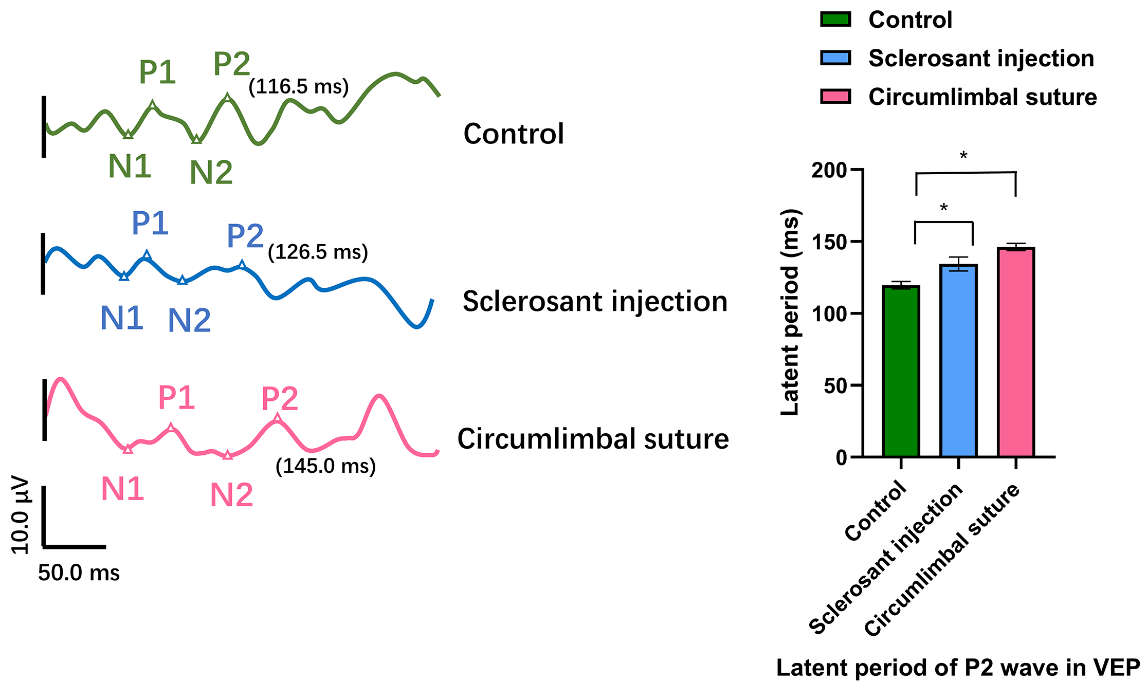

Supplement: Supplementary file 6 — Additional file 6: Figure S5. Results of electrophysiology in ocular hypertension (OHT) eyes [file 40662_2022_319_MOESM6_ESM.docx]
